# DAta Linkage to Enhance Cancer Care (DaLECC): Protocol of a Large Australian Data Linkage Study

**DOI:** 10.3390/ijerph20115987

**Published:** 2023-05-29

**Authors:** Laura C. Edney, Jackie Roseleur, Tim Bright, David I. Watson, Gaston Arnolda, Jeffrey Braithwaite, Geoffrey P. Delaney, Winston Liauw, Rebecca Mitchell, Jonathan Karnon

**Affiliations:** 1 Flinders Health and Medical Research Institute, Flinders University, Bedford Park, SA 5042, Australia; 2 Oesophagogastric Surgery Unit, Flinders Medical Centre, Bedford Park, SA 5042, Australia; 3 College of Medicine and Public Health, Flinders University, Bedford Park, SA 5042, Australia; 4 Australian Institute of Health Innovation, Macquarie University, North Ryde, NSW 2109, Australia; 5 Liverpool Cancer Therapy Centre, Liverpool, NSW 2170, Australia; 6 South Western Sydney Clinical School, University of New South Wales, Liverpool, NSW 2170, Australia; 7 Ingham Institute for Applied Medical Research, Liverpool, NSW 2170, Australia; 8 St. George Cancer Care Centre, St. George Hospital, Kogarah, NSW 2217, Australia; 9 St. George Hospital Clinical School, University of New South Wales, Sydney, NSW 2217, Australia

**Keywords:** cancer, survivorship, economic evaluation, cost analysis, cost effectiveness, economic burden, patient outcomes, health services research, quality of life, comorbidities, primary care

## Abstract

Cancer is a leading cause of global morbidity and mortality, accounting for 250 Disability-Adjusted Life Years and 10 million deaths in 2019. Minimising unwarranted variation and ensuring appropriate cost-effective treatment across primary and tertiary care to improve health outcomes is a key health priority. There are few studies that have used linked data to explore healthcare utilisation prior to diagnosis in addition to post-diagnosis patterns of care. This protocol outlines the aims of the DaLECC project and key methodological features of the linked dataset. The primary aim of this project is to explore predictors of variations in pre- and post-cancer diagnosis care, and to explore the economic and health impact of any variation. The cohort of patients includes all South Australian residents diagnosed with cancer between 2011 and 2020, who were recorded on the South Australian Cancer Registry. These cancer registry records are being linked with state and national healthcare databases to capture health service utilisation and costs for a minimum of one-year prior to diagnosis and to a maximum of 10 years post-diagnosis. Healthcare utilisation includes state databases for inpatient separations and emergency department presentations and national databases for Medicare services and pharmaceuticals. Our results will identify barriers to timely receipt of care, estimate the impact of variations in the use of health care, and provide evidence to support interventions to improve health outcomes to inform national and local decisions to enhance the access and uptake of health care services.

## 1. Introduction

Cancer is a leading cause of morbidity and mortality globally, accounting for 250 Disability-Adjusted Life Years and 10 million deaths in 2019 [1], and is estimated to cost the world economy INT $25.2 trillion between 2020 and 2050 [2]. Appropriate, cost-effective treatment to improve health outcomes is therefore a key global health priority. Minimising unwarranted variation and ensuring that health care meets the recommended guidelines for all patients both within and between cancer types can contribute to improving overall health outcomes and ensuring that these health outcomes are equitably distributed across the population through appropriate distribution of health funds [3]. Spending on cancer and other neoplasms accounted for 8.6% of the total disease expenditure in 2015–16, estimated at AUD10.1 billion with the majority of this (AUD9.7 billion) directed toward the diagnosis and treatment of cancer, with AUD409 million spent on national bowel, breast, and cervical screening programs [4].

Linked health systems data provide a basis for population-level analyses of pathways of care, costs, and outcomes to identify emerging trends as well as important areas of variation, for example, based on geographic or demographic factors [5,6]. Such analyses have the potential to inform decision-making across a health system, including health policy decisions around funding mechanisms through to local health service decisions to improve service delivery for specific patient groups. Despite this, analyses of linked datasets often exclude consideration of cost; for example, a recent systematic review of systemic therapies for breast cancer patients explored in claims data internationally found that only 22% of 106 therapies included a consideration of healthcare costs [7].

In Australia, a range of linked datasets have been assembled and analysed with a focus on cancer [8,9,10,11,12], but the reported analyses of these datasets have had less focus on primary health care or pre-diagnosis patterns of care. Primary health care is an important component of care pathways for cancer patients both before and after a cancer diagnosis [13].

Cancer diagnoses can occur through different pathways, including general practice referrals, screening programs, and emergency department (ED) presentations. ED presentations are an independent predictor of lower survival, even after adjusting for stage at diagnosis [14]. Population-level analyses of the patterns of primary health care use and clinical, geographical, and demographic factors have the potential to inform actions to reduce diagnostic pathways that include the ED.

Cancer survivors are more likely to have mental and physical health comorbidities than non-cancer patients [15]. The management of existing comorbidities has been shown to decline after a cancer diagnosis as both patients and clinicians focus on the cancer, placing patients at risk of poorer outcomes [16,17]. Patients are also at increased risk of developing new physical comorbidities as side effects of cancer therapy, examples of which include arthritis, hypertension and osteoporosis in breast cancer patients [18], and diabetes in prostate cancer patients [19]. More generally, cancer patients have high rates of unmet supportive care needs that have significant emotional and psychological impacts [20].

The DAta Linkage to Enhance Cancer Care (DaLECC) study will link cancer registry data with data describing health care use in primary and tertiary care settings, including up to ten years of pre-cancer diagnosis data. The pre-diagnosis data will facilitate analyses of care pathways to diagnosis as well as the more accurate estimation of pre-existing comorbidities.

### Research Aims

This research aims to answer a range of questions related to understanding variation in healthcare use both pre- and post-diagnosis, and the impact of this variation on health outcomes and costs. We outline our focus on four key broad areas that will be applied across cancer types: diagnosis through ED, regularity of general practitioner (GP) care pre- and post-diagnosis, time to treatment, and understanding patients’ supportive care needs post-diagnosis. We additionally provide a case study example of the aims for one specific cancer type, upper gastrointestinal (GI) cancer, for which we have additional information from a clinical database. Specific research aims and methods to address these are outlined in the Data Analyses section.

## 2. Materials and Methods

### 2.1. Cohort

**Figure 1:**
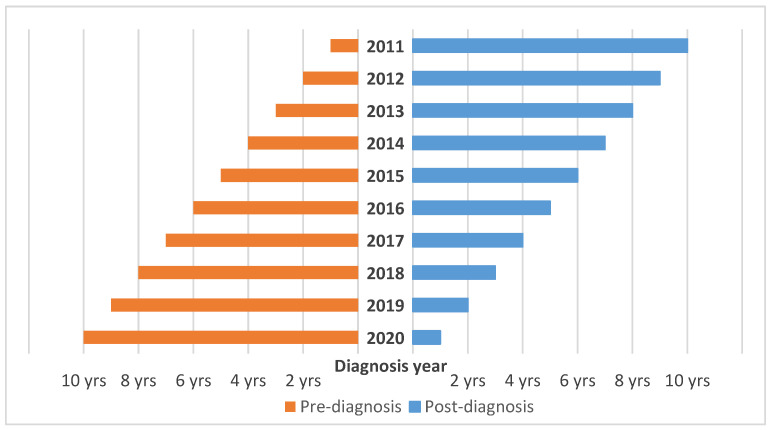
Pre- and post-diagnosis data available by year of cancer diagnosis.

The study cohort will be derived from the South Australian Cancer Registry (SACR). Cancer is a legally notifiable disease in all states and territories in Australia, requiring the reporting of all cancers except basal and squamous cell skin carcinomas [21]. All South Australian (SA) residents aged 18 and older diagnosed with cancer, excluding non-melanoma skin cancer, between 1 January 2011 and the latest available data, which is estimated to be 31 December 2020, will be eligible for inclusion in the study. Healthcare utilisation data (i.e., hospital inpatient and ED data, and Medicare Benefits Schedule (MBS) and Pharmaceutical Benefits Scheme (PBS) data) were requested from 1 January 2010 for each identified patient. All patients will therefore have at least one year of pre-diagnosis data available and between one and ten years of post-diagnosis data depending on the year of cancer diagnosis, as depicted in Figure 1. A study sample size of approximately 103,000 cancer patients is expected. SA is the fourth largest of Australia’s states and territories (984,321 square kilometers) [22] with a population of 1.8 million [23] and three-quarters of residents living in metropolitan Adelaide [24].

### 2.2. Data Linkage

**Figure 2:**
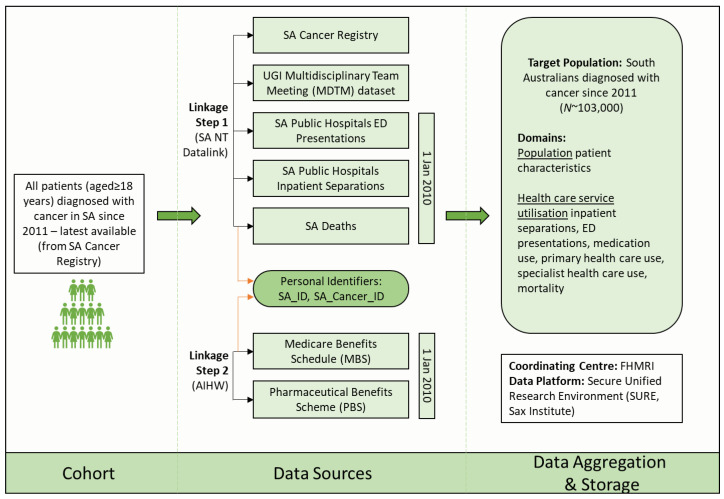
Framework for DaLECC project describing datasets and data linkage process. AIHW, Australian Institute of Health and Welfare; ED, Emergency Department; FHMRI, Flinders Health and Medical Research Institute; GI, Gastrointestinal; MBS, Medicare Benefits Schedule; PBS, Pharmaceutical Benefits Scheme; SA, South Australia; SURE, Secure Unified Research Environment.

SA NT DataLink is part of the Population Health Research Network (PHRN), an Australian wide national data linkage network, where SA NT DataLink is responsible for data linkage for South Australian and Northern Territory datasets [25]. SA NT DataLink will extract the study cohort from the SACR, link to SA Public Hospital inpatient separations, SA Public Hospital ED presentations, SA Deaths, and the Upper GI Multidisciplinary Team (MDT) database for a subset of patients diagnosed with upper GI cancer. Once completed, the requested content data will be uploaded to the Secure Unified Research Environment (SURE), SAX Institute, with a new project-specific person ID called SA_ID. Thereafter, SA NT DataLink will send the cohort personal identifiers to the Australian Institute of Health and Welfare (AIHW) with the SA_ID and the SA_Cancer_ID (Figure 2). SA_Cancer_ID was previously provided to AIHW with SA data to be loaded to the Australian Cancer Database and may assist with the linkage. Once received, AIHW will link the cohort to the Commonwealth MBS and PBS dataset. Once completed, the requested content data will be uploaded into SURE with the SA_ID. Under no circumstances will the researchers have access to the file that links the personal information of individuals to the SA_ID. This is controlled by SA NT DataLink with strict data controls. All data transfers will involve encrypted files sent via a secure messenger service. All unit record data will be stored and accessed through SURE [26]. Any summary data approved for release through the SURE curated gateway will be stored at Flinders University on password-protected computers.

Details of the datasets to be linked to the SACR are as follows:

### 2.3. Data

#### 2.3.1. South Australian Cancer Registry (SACR)

All cases of invasive cancer are notifiable under the Cancer Reporting Regulations under the *Health Care Act 2008* [27] by pathology laboratories and health care institutions. Most notifications are received electronically by the SACR, managed by Wellbeing SA. A range of quality assurance measures are in place, including electronic notification from multiple sources, follow-up to locate any missing pathology reports, annual internal identification, removal of duplicate individuals, and real time validation checks on sex/cancer, morphology/topography, and age/cancer combinations [28]. The full data item list for extracted variables from the SACR is available in Appendix A.

#### 2.3.2. South Australian Upper Gastrointestinal (GI) Multidisciplinary Team (MDT) Database

This is a prospectively maintained database designed to support the SA Statewide Upper GI MDT which manages over 90% of new upper GI presentations in SA, and some NT presentations. The full data item list for extracted variables from the SA Upper GI MDT database is available in Appendix A.

#### 2.3.3. Healthcare Utilisation

The Australian healthcare system is funded through private and public funding with Medicare, the universal tax-funded health insurance providing citizens and permanent residents with free public hospital treatment and subsidised healthcare services through the MBS and pharmaceuticals through the PBS.

##### MBS and PBS

The MBS and PBS are administrative claims datasets. The MBS includes the utilisation and costs of subsidised out-of-hospital services for primary care, including GP and specialist visits, pathology and imaging services, and visits to allied health professionals and Medicare rebates for private patients admitted to public hospitals. MBS claims data exclude the Department of Veteran’s Affairs beneficiaries and information on public patients in public hospitals, captured through the SA inpatient dataset. The PBS includes the utilisation and costs of subsidised pharmaceuticals listed on the PBS, excluding private prescriptions, over-the-counter medications, Repatriation Pharmaceutical Benefits Scheme prescriptions, and any pharmaceuticals dispensed under special arrangements. Costs incurred will be analysed from both the healthcare perspective, based on the recorded government benefit paid for the MBS and PBS, and from a societal perspective, including individual out-of-pocket spending based on the difference between the fee charged and the benefit paid for the MBS, and on the patient contribution for the PBS. Services will be recorded to their date of service for the MBS and to their date of supply for the PBS. The full data item list for extracted variables from the MBS and PBS is available in Appendix A.

##### Hospital Care

Hospital care is captured through inpatient separations and ED presentations. Inpatient separations, including completed admitted episodes of care due to discharge, transfer, or death, are recorded to Australian refined diagnosis-related group (AR-DRGs) codes which are used to assign associated costs based on yearly price weights, adjustment factors, and the National Efficient Price (NEP). ED presentations are recorded to Urgency Related Groups (URGs) derived from the type of visit (emergency, trauma, planned review, unplanned review, planned admission, patient in transit, other, unknown), episode and status (admitted, non-admitted, did not wait for attendance, left at own risk, died in ED, dead on arrival, referred to another health service, not stated), triage category (resuscitation: immediate, emergency, urgent, semi-urgent, non-urgent, not assigned), and major diagnostic block (International Classification of Diseases, Tenth Revision (ICD-10)), with the costs assigned based on yearly price weights, adjustment factors, and the NEP. The full data item list for extracted variables from inpatient separations and ED presentations is available in Appendix A.

Limitations associated with the administrative nature of these datasets are reported in Table 1.

### 2.4. Variables

The variables extracted from each database are described in Appendix A. Additional derived variables include:

#### 2.4.1. Distance to Healthcare

Travel distance for hospital and Medicare attendances will be estimated for periods of time pre- and post-diagnosis. The most frequent pharmacy postcode for PBS scripts serviced in a given year will be employed as a proxy for the patient postcode in that year and used to calculate the distance to inpatient separations and ED presentations based on the postcode of the organisational identifier, to MBS services based on the servicing provider postcode, and for PBS scripts based on the prescriber postcode. Distance estimates will be multiplied by the Australia Tax Office cents per kilometer formula to estimate the car expenses associated with travel for healthcare.

#### 2.4.2. GP Regularity

The GP regularity index [29,30] will be used to capture the dispersion of GP contacts independent of frequency, where regular distribution over time is assumed to indicate planned rather than reactive care [31]. The index (*R*) ranges from 0 (least regular) to 1, calculated as *R* = 1/1 + (coefficient of variation (days between GP contacts)) [29,30]. GP contacts will be captured from MBS claims for attendances by GPs, capturing all GP attendances in Australia and presented for patients prior to and following diagnosis.

#### 2.4.3. Comorbidities

Comorbidities will be estimated using the Cancer, Care, and Comorbidity (C3) index [32] and the pharmacy-based comorbidity index (PBCI) [33]. These indices calculate comorbidity index scores based on the association of conditions with non-cancer mortality among cancer patients. The C3 index weights 42 chronic conditions identified from inpatient separations [32] and the PBCI weights 19 conditions derived from pharmaceutical data [33]. Total individual index scores will be calculated as the sum of weights for all conditions identified for each individual for the C3 and PBCI indices in both the pre- and post-diagnosis time periods. Two indices are used as hospitalisation-based data only capture comorbidities among hospitalised patients, whereas pharmaceutical data allow for the identification of comorbidities among patients who are not hospitalised. This is particularly relevant in younger patient populations, such as those with breast cancer who have less frequent hospitalisations [33].

#### 2.4.4. Diagnosed with Cancer through the ED

Cancer diagnosis from the SACR recorded within 30 days following an emergency hospital admission, independent of clinical reason [34]. Additional cut-off periods of 60 and 90 days will be explored, and increased specificity through restricting the definition to include emergency hospitalisations without an elective hospital admission through to diagnosis date, as in McPhail et al. [34].

#### 2.4.5. Supportive Care

The presence of additional comorbidities identified in the post-diagnosis period that were not present in the pre-diagnosis period will be reviewed by time since diagnosis by clinician authors. The conditions considered more likely to be identified during cancer treatment rather than as a result of cancer diagnosis or treatment will be excluded. The remaining additional comorbidities in the post-diagnosis period will be assumed to be the result of cancer diagnosis or treatment and classified as a supportive care need. These additional comorbidities will be identified from inpatient separations based on the C3 index, the PBCI, the categories of PBS scripts dispensed, such as prescriptions for antihypertensives or for psychoanaleptics, and specific MBS claims, such as GP visits for a mental health care plan. This captures variation in the use of services for specific categories of supportive care, and may thus represent an underestimate of true supportive care needs where treatment is not accessed. Out-of-pocket spending on medical services from the MBS and on pharmaceuticals from the PBS will be combined with financial costs associated with travel time to provide an indication of financial burden as an additional supportive care need.

### 2.5. Ethics

Ethics approval was obtained from the SA Department of Health and Wellbeing Human Research Ethics Committee (HREC Approval 2021/HRE00069) and from the AIHW Human Research Ethics Committee (EO2021-1-1238). A waiver of consent was sought and granted by the AIHW Human Research Ethics Committee pursuant to s.95 of the Privacy Act 1988 (Commonwealth). Patient privacy will be protected as unique identifiers will not be shared with researchers and authors. Only SA data custodians and linkage officers from SA NT DataLink and AIHW will have access to personal information. All de-identified unit record data will be stored in SURE, independently managed by the SAX Institute [26].

### 2.6. Research Aims

#### 2.6.1. Pathways to Diagnosis

1. Describe diagnosis pathway through ED by cancer type and year
2. Evaluate the predictors of variation in ED diagnosis pathway
3. Evaluate whether ED diagnosis pathways predict all-cause mortality and treatment costs

#### 2.6.2. Regularity of GP Care

1. Describe GP regularity pre- and post-diagnosis both within individuals and between cancer types
2. Evaluate the predictors of variation in GP regularity pre- and post- diagnosis
3. Evaluate whether GP regularity pre- and post-diagnosis predicts all-cause mortality and health costs, and additionally whether GP regularity pre-diagnosis predicts the extent of the disease at diagnosis

#### 2.6.3. Time to Treatment from Diagnosis

1. Describe the proportion of the cohort who receive treatment following diagnosis in the time frames suggested by Optimal Care Pathway (OCP) guidelines for each cancer type, and whether this varies by cancer type and year
2. Evaluate the predictors of variation in time to treatment
3. Evaluate whether time to treatment predicts all-cause mortality and health costs

#### 2.6.4. Supportive Care Needs

1. Describe supportive care needs and patterns of supportive care experienced by patients’ post-diagnosis by cancer type
2. Evaluate predictors of variation in supportive care needs and supportive care
3. Evaluate whether variation in supportive care needs and supportive care predicts all-cause mortality and health costs

#### 2.6.5. Case study: Upper GI Cancer

1. Describe MDTs and change over time in the number of patients discussed, rate of claiming MBS MDT item numbers, number of diagnostic tests completed pre- and post-MDT, time between diagnosis and MDT discussion, and number of MDT discussions per patient
2. Evaluate whether MDT attendance has an impact on patterns of care, including inpatient separations, ED presentations, GP attendances, allied health, overall all-cause mortality, and health costs
3. Explore the use of real-world evidence to evaluate pharmaceuticals, such as Fluorouracil, Leucovorin, Oxaliplatin, and Docetaxel (FLOT) chemotherapy and Herceptin, and the use of new healthcare services, including the provision of regional chemotherapy services.

### 2.7. Data Analyses

#### 2.7.1. Descriptive Statistics

Descriptive statistics will be used to:

Describe the proportion of patients who were diagnosed with cancer through the ED by cancer type and across years, and to describe the frequency and type of healthcare utilisation (including the total number of healthcare services, subcategories for inpatient separations, ED presentations, and GP attendances) and their associated costs in the 1 year and 6 months prior to diagnosis for those diagnosed through the ED, compared to those not diagnosed through the ED. 

Describe mean GP regularity contact for 1-year periods before and after diagnosis for individuals and between cancer types in the post-diagnosis time period, and examine the relationship between GP regularity and the rates and costs of inpatient separations and ED presentations. 

Describe mean time to treatment by cancer type, the proportion of patients receiving treatment within the time frames recommended by the OCP guidelines for cancer types with an OCP guideline, and the associated healthcare utilisation rates and costs (including total healthcare services, inpatient separations, and ED presentations) in 1-year periods post-diagnosis.

Describe the range and number of supportive care needs and supportive care following patients’ cancer diagnosis and describe the associated healthcare utilisation and costs (including for total healthcare services, inpatient separations, ED presentations, GP attendances, and categories of pharmaceutical claims) by frequency of supportive care and categories of supportive care needs.

#### 2.7.2. Predictors of Variation in Care

For binary outcome models (i.e., diagnosis through ED and time to treatment meeting OCP guidelines), multiple logistic regression models will be used to explore the impact of a range of predictors, including patient characteristics (age, gender, insurance status, location), clinical characteristics (number of comorbidities, cancer type, and disease extent at diagnosis), year, and GP regularity prior to diagnosis on the likelihood of being diagnosed through the ED and receiving treatment within OCP guideline recommendations. The results will be presented as odds ratios (OR) with 95% confidence intervals (CIs).

For continuous outcome models (i.e., GP regularity pre-diagnosis, GP regularity post-diagnosis, and supportive care), bivariate linear regression models will be used to evaluate the relationships between (1) GP regularity prior to diagnosis with patient characteristics (age, gender, insurance status, location), year, and clinical characteristics (comorbidities), (2) GP regularity following cancer diagnosis with patient characteristics (age, gender, insurance status, location), clinical characteristics (number of comorbidities, cancer type, and disease extent at diagnosis), and year, and (3) supportive care with patient characteristics (age, gender, insurance status, and location), year, and clinical characteristics (comorbidities, cancer type, disease extent at diagnosis, and GP regularity).

#### 2.7.3. Consequences of Variation in Care

Primary outcome measures for all models will include (1) costs presented in AUD2020 associated with total healthcare use and subcategories of healthcare use, including for inpatient separations, ED presentations, and claims for GP attendances, other medical services, and categories of pharmaceutical claims, and (2) health outcomes captured through all-cause and cancer-specific mortality, supplemented with Health-Related Quality-of-Life (HRQoL) estimates from the literature where available.

Separate two-part models will be used to explore the impact of an ED diagnosis, GP regularity pre- and post-diagnosis, time to treatment within OCP guidelines, and the number of supportive care needs on healthcare costs, where probit models are used to predict the probability of incurring costs, followed by Generalised Linear Models (GLM) with log-link and gamma distributions, in order to model healthcare costs, conditional on any healthcare costs. Relevant covariates will include patient and clinical characteristics. 

Kaplan-Meier survival analyses will be used to compare all-cause survival rates based on the average values of continuous predictors (i.e., GP regularity pre- and post-diagnosis and the number of supportive care needs) and for those with an ED diagnosis compared to those without, and for those who received treatment within OCP guidelines compared to those who received treatment outside of these guidelines. Censored patients will be assumed to be alive at the end of the study period. Cox proportional hazard analyses will be used to estimate hazard ratios for the association between the risk of all-cause mortality and the predictors adjusted for patient and clinical characteristics and year. Hazard ratios and their 95% Cis will be presented. Competing risks regressions will be used to identify independent predictors of cancer-related and non-cancer-related mortality adjusted for patient and clinical characteristics, with subhazard ratios (SHRs) and their associated 95% Cis presented. 

The cost-effectiveness of time to treatment meeting OCP guidelines (e.g., treatment to commence within 2 weeks of MDT) will also be explored using a local instrumental variable (LIV) approach to address unmeasured confounding (e.g., due to baseline prognosis measures, such as disease severity) and heterogeneity (e.g., due to patient and contextual factors) in our observational data. Individual-level treatment effects of OCP adherence on the costs and outcomes estimated from the LIV approach (i.e., person-centered treatment (PeT) effects) will be used to generate mean treatment effect parameters (i.e., effect on treated (TT), effect on untreated (TUT), and average treatment effect (ATE) [35]), and the results will be aggregated over subgroups with different expectations of relative effectiveness and cost-effectiveness (e.g., by cancer type). The results will be presented as net health benefits and will include an estimation of the potential national benefits and cost impact if all patients were to adhere to the specific OCPs for time to treatment. 

#### 2.7.4. Case Study: Upper GI Cancer

The characteristics of the MDT meetings will be described, including the proportion of patients diagnosed with an upper GI cancer in SA who were discussed at the MDT, the rate of MBS claims for an MDT, the average time between diagnosis and the MDT discussion, and the frequency with which each patient is discussed at an MDT, and how these characteristics have changed over time. 

A LIV approach will again be employed to evaluate the cost-effectiveness of being discussed at an MDT. The results will be presented as net health benefits and will include an estimation of the national benefit and cost impact if all upper GI patients were discussed at an MDT.

The use of interrupted time series methods will be employed to explore change over time in both costs and outcomes where new technologies (e.g., pharmaceuticals including FLOT chemotherapy or Herceptin, or the provision of new healthcare services including regional chemotherapy services) were introduced into practice at a specific time point. ITS will control for covariates at the population level, and cost and mortality data will be compiled by months. Point estimates for a step-change at the time of technology listing and a change in trend will be presented, and any presence of seasonality in the estimates will be adjusted for. All statistical analyses will be performed in STATA [36] or R [37].

## 3. Discussion

This paper has described the DAta Linkage to Enhance Cancer Care (DaLECC) dataset and planned health economics-focused analyses to inform potential improvements in the organisation and delivery of cancer care in SA, with potential generalisability to the rest of Australia. This protocol provides enough detail that equivalent summary variables could be generated based on distinct data items from linked administrative datasets internationally. The linkage of health systems data to cancer registry data in Australia is not novel, but the proposed analyses are distinct from previous health economics-focused analyses of cancer care.

Similar linked datasets have been assembled in other Australian states, including two using data from Queensland [CancerCostMod [8]; COS-Q [11]]. Both datasets linked public hospital ED and inpatient data and MBS and PBS data to cancer registry data. The dataset reported by Callander and colleagues has primarily been used to report on the costs of cancer care, with a focus on patient co-payments and cost differences, e.g., by Indigenous status, rurality, and socioeconomic status [38]. The dataset reported by Merollini et al. has also been used to estimate the costs of cancer care, focusing on costs by time since cancer diagnosis including data up to 20 years post-diagnosis [39]. The protocol also refers to analyses of variation in health service use and costs, lifetime models of costs by cancer type, and analyses of opportunities to improve service delivery. In NSW, the 45 and Up study linked the same data sources to survey data collected from 7624 study participants who were diagnosed with cancer and up to 3 control participants [9]. These data have also been used to estimate cancer care costs by cancer type and phase of care (initial, continuing, and terminal). 

In SA, previous linked data projects have addressed a range of questions, including concordance with treatment guidelines [40], variation in healthcare expenditure [41], use of colonoscopy pre-colorectal cancer diagnosis [10], and screening-treatment-mortality pathways [42]. A Cancer Data and Aboriginal Disparities (CanDAD) linked dataset was also established, comprising all Aboriginal people living in SA at the time of their cancer diagnosis between 1990 and 2010, and one matched non-Aboriginal person with cancer for each Aboriginal member of the cohort [12]. A unique aspect of this dataset was the manual staging of each cohort member’s diagnosed cancer. The published analyses of this dataset have quantified disparities in access to care and health outcomes between Aboriginal and non-Aboriginal people with cancer. 

There are some similarities between the planned analyses for the DaLECC dataset and some of the previous analyses, such as the analysis of pre-diagnostic healthcare use. However, previous analyses have had little focus on primary health care. The planned analyses will describe variation in the use of primary health care, both pre- and post-diagnosis, including the use of government-subsidised general practice and allied health consults and community pharmacy supplied medications. Going beyond the description of variation, a general aim is the estimation of the expected value of opportunities for improving care for cancer patients. For example, analyses of geographic, demographic, and clinical characteristics, pre-cancer patterns of primary care use, and cancer stage at diagnosis or ED-based cancer diagnoses could inform analyses of the value of targeted awareness-raising campaigns. Likewise, the associations between patterns of primary care use and post-cancer diagnosis inpatient separations for non-cancer related conditions could form the basis for estimating the value of promoting the primary care-based management of supportive care needs.

For patients diagnosed with an upper GI cancer, an extended linked dataset will be analysed that includes data collected by the clinical team managing the statewide multi-disciplinary team meetings. The foci of the planned analyses include comparisons of the pathways, costs, and outcomes for patients who were discussed and not discussed at an MDT, and of the associations between MDT actions and costs and outcomes. This is a novel dataset that provides a case study of the value of linking MDT data, where similar data is likely collected by the hundreds of MDTs that meet across Australia.

### 3.1. Stakeholder Engagement

Stakeholder engagement is essential for generating evidence with a meaningful impact. Cancer Voices SA is a consumer organisation focused on advocacy, awareness raising, and information sharing [43]. It also supports consumer representation on cancer-related research projects through its Consumer Involvement in Research Program. The DaLECC project will engage with Cancer Voices SA to appoint consumer representatives to provide the consumer perspective on the proposed research. Additionally, consumer perspectives will be sought from the Flinders Health and Medical Research Institute (FHMRI) Consumer Advisory Board. Consumers will inform the interpretation of the results, particularly considering the limitations in the data. As the DaLECC project aims to identify supportive care sought by patients, consumers will provide input on the importance of these findings and will advise on the services available to address supportive care needs. Furthermore, consumer perspectives will inform the potential next steps of the project and support our dissemination strategies to relevant stakeholders by reviewing plain English summaries of the research findings to be used in the translation of research outputs for general media articles, including through The Conversation and Flinders University website and social media channels. Study findings will also be disseminated at national and international conferences, in peer-reviewed publications, and locally through primary health networks and local health networks, particularly findings that can support local health service planning. 

In addition to consumer engagement, the DaLECC project will continue to work closely with clinicians. Clinician engagement ensures that the research aims are relevant to clinical practice by identifying key clinical needs and gaps. It also facilitates the interpretation of findings to the local clinical context and can increase the likelihood of implementation of future interventions into practice.

### 3.2. Limitations

There are limitations to the DaLECC dataset that limit its ability to address certain research questions, as outlined in Table 1. The lack of complete staging information on diagnosed cancers restricts comparisons of pre-diagnostic pathways to differences in the likelihood of being diagnosed with early- versus late-stage cancer, but this is still a meaningful differentiation. The lack of staging data also limits the extent to which adherence to clinical guidelines can be assessed, although this is not a focus of the planned analyses. No formal recording of cancer progression is also a limitation, although analyses of cancer-related healthcare use will be undertaken to develop a proxy for cancer progression with a focus on identifying progression from early- to late-stage cancer. This is a sufficient outcome for the planned analyses, given that the analysis of detailed treatment pathways is not a focus.

The exclusion of data describing outpatient clinic attendances at public hospitals is a significant limitation with respect to describing care received post-diagnosis. This omission is expected to reduce the likelihood of identifying significant associations between the use of primary care and non-cancer hospital encounters, as patients not using primary care may be attending outpatient clinics. No direct information on reasons for GP consultations restricts the specificity of analyses with respect to the use of primary healthcare. However, pharmaceutical use can be analysed to inform the management of different clinical issues, and there are some MBS items for specific conditions, including mental health and diabetes. Changes in the use of other MBS items, such as management plans and team care arrangements, will also be informative. 

Patient co-payments are recorded for MBS and PBS items, for which adjustments for the Medicare safety net can be made. However, other potential costs, including non-Medicare-subsidised health care, insurance covered costs, and lost income for patients and carers, are not recorded; hence, the planned analyses do not focus on patient costs. Patient specific cost estimates for care received at public hospitals are not available, and so average costs will be estimated for ED presentations and inpatient admissions using activity-based funding price weights for different urgency- and diagnosis-related groups. This reduces the precision of the costing, but is not expected to have a meaningful impact on the interpretation of the outputs of the planned analyses.

## 4. Conclusions

Improving cancer services is a key global health priority. Evidence suggests that there are wide variations in health outcomes for cancer patients [44,45], although the extent to which this is due to variations in the access and use of health care is unclear [46]. This research seeks to establish methods to identify and estimate the impact of variations in the use of health care and to inform national and local decisions around improving access to health care. While the conclusions are relevant for SA, the methods are applicable beyond this. A key strength of this study is the inclusion of population-level data for all cancer types, which facilitates complete full analyses of the geographic, demographic, and clinical factors associated with potential variations in the access and use of health care, and their consequences. 

## Figures and Tables

**Table 1:** Data limitations.

Data Limitations	Response to Limitations
Medicare	
No clinical information is recorded to Medicare.	Informed assumptions regarding reasons for selected pharmaceutical prescriptions and medical services can be made.
Some privately funded health services not captured.	Our focus is on the public healthcare system; however, interpretation of post-diagnosis pathways of care will also need to consider the omission of privately funded allied health services.
Medicare, PBS	
Government contribution may be lower than recorded if a medication has a special pricing arrangement or price volume agreement.	A list of scripts subject to these agreements will be compiled to inform the extent to which the benefit paid may be overestimated.
Medications supplied through the remote area Aboriginal Health Services are not captured.	Omission will be considered when interpreting data, e.g., for patients living in remote areas.
Over-the-counter medications are not captured.	Over-the-counter medications generally represent lower-cost medications, but their omission will be considered when interpreting data.
SA public hospital data	
Individual-level cost data not available.	Costs can be generated from AR-DRG and URG codes for inpatient separations and ED presentations, respectively.
Outpatient clinic attendance data not available.	Omission will be considered when interpreting post-diagnosis pathways of care.
SACR	
Staging data not recorded.	Early- and late-stage diagnoses can be defined.
Cancer recurrence not recorded.	Time to progression to late stage will be estimated.

AR-DRG, Australian refined diagnosis-related group; ED, Emergency Department; PBS, Pharmaceutical Benefits Scheme; SACR, South Australian Cancer Registry; URG, Urgency Related Groups.

## Data Availability

Not applicable.

## Appendix A

**Table A1:** Full list of linked variables from each dataset.

Database	Variables
Study cohort	
SA Cancer Registry (SACR)	Patient data Age at diagnosis Date of birth Gender Date of death Age at death Socio-Economic Indexes for Areas (SEIFA) Australian Standard Geographical Classification (ASGC) Remoteness Areas classification Disease characteristics Date of diagnosis Cancer site/type (ICD-10) Most valid basis of diagnosis Staging (disease extent) Topography (ICD-O-3) Morphology (ICD-O-3) Underlying cause of death
Healthcare utilisation and costs	
SA public hospital inpatient separations	Admission date Admission urgency status Condition onset flag Intended length of stay Length of ICU stay Number of days of hospital-in-the-home care Patient election status Procedure code (ACHI 9th ed) Separation date Principal diagnosis (ICD-10-AM) Additional diagnoses (ICD-10-AM) Source of funding Geographical remoteness Organisation identifier Region identifier Sector Care type Hospital insurance status Patient—previous specialised treatment Medicare eligibility status Australian refined diagnosis-related group (AR-DRG)
SA hospital emergency department (ED) presentations	Principle diagnosis Diagnosis classification Additional diagnosis code Presentation date Physical departure date Type of visit to ED Urgency related group (URG) Funding eligibility indicator Organisation identifier Clinical care commencement date Episode and date Episode and status Service episode length Triage category Triage date Compensable status
Medicare Benefits Scheme (MBS)	Servicing provider postcode Referring provider postcode Servicing provider number Servicing provider practice number Servicing provider registered speciality Number of services Referring provider number Referring provider practice number Referring date Date of service Date of processing Current item number Original item number at date of service Hospital flag Bulk-billing flag MBS category code MBS group code MBS subgroup code Broad type of service Benefit paid amount Fee charged amount Schedule fee amount
Pharmaceutical Benefits Scheme (PBS)	Pharmacy postcode Prescriber postcode PBS item Drug type Benefit amount Patient contribution amount Streamlined authority approval number Under co-payment prescription type CTG co-payment eligibility code Pharmacy approval type Pharmacy identifier Prescriber speciality Prescriber type Date of prescribing Prescriber identifier Repeat prescription indicator Number of scripts dispensed Quantity supplied Date of supply Patient category
Other	
SA Deaths	Date of death Underlying cause of death Other causes of death
Upper Gastrointestinal (GI) Multidisciplinary Team meeting (MDT) database	Hospital identifier Cancer site Date of MDT Meeting Number of times the patient has previously been discussed at an MDT Flag for cancer recurrence Flag for whether a treatment decision was made Reason for why treatment decision not made Treatment recommendation

ACH, Australian Classification of Health Interventions; AR-DRG, Australian refined diagnosis-related group; ASGC, Australian Standard Geographical Classification; CTG, Closing the Gap; ED, Emergency Department; GI, Gastrointestinal; ICD-10, International Classification of Diseases 10th Revision; ICD-10-AM, International Classification of Diseases 10th Revision, Australian Modification; ICD-O-3, International Classification of Diseases for Oncology, third edition; ICU, Intensive care units; MBS, Medicare Benefits Schedule; MDT, Multidisciplinary Team; PBS, Pharmaceutical Benefits Scheme; SEIFA, Socio-Economic Indexes for Areas; URG, Urgency Related Groups.
